# IL-6 Reduces Mitochondrial Replication, and IL-6 Receptors Reduce Chronic Inflammation in NAFLD and Type 2 Diabetes

**DOI:** 10.3390/ijms22041774

**Published:** 2021-02-10

**Authors:** Daria Skuratovskaia, Aleksandra Komar, Maria Vulf, Hung Vu Quang, Egor Shunkin, Larisa Volkova, Natalia Gazatova, Pavel Zatolokin, Larisa Litvinova

**Affiliations:** 1 Center for Immunology and Cellular Biotechnology, Immanuel Kant Baltic Federal University, 236041 Kaliningrad, Russia; alexandkomar@gmail.com (A.K.); mary-jean@yandex.ru (M.V.); hungrgmu@gmail.com (H.V.Q.); egor.shunkin@gmail.com (E.S.); n_gazatova@mail.ru (N.G.); endozapa@yandex.ru (P.Z.); larisalitvinova@yandex.ru (L.L.); 2 Diagnostics Laboratory, Immanuel Kant Baltic Federal University, 236041 Kaliningrad, Russia; volkova-lr@rambler.ru

**Keywords:** IL-6, IL-11, gp130/sIL-6Rb, sIL-6Ra, obesity, NAFLD, mitochondria, T2DM

## Abstract

Interleukin (IL)-6 family cytokines act through a receptor complex with gp130 subunits. IL-6 is a pleiotropic cytokine that regulates inflammation and liver regeneration. Mitochondria are the first to respond to stress and adapt their dynamics in conditions of damage. In this regard, the study aimed to investigate the role of the IL-6 cytokine family (sIL-6Ra, gp130/sIL-6Rb, and IL-11) in the regulation of mitochondrial dynamics in the liver in obese patients and to assess the contribution of these cytokines to the pathogenesis of type 2 diabetes mellitus (T2DM). We studied 134 obese patients with and without T2DM and 41 healthy donors. We found that increasing the concentration of sIL-6Ra and gp130/sIL-6Rb protected against carbohydrate disorders in obese patients and prevented non-alcoholic fatty liver disease (NAFLD) progression in obese patients. An increase in plasma IL-6 levels is associated with decreased, mitochondrial transcription factor A (TFAM) protein production in liver biopsies in obese patients with and without T2DM. Replication, transcription, and division processes in liver biopsy were reduced in patients with T2DM. Inflammatory processes stimulate liver cell apoptosis in obese patients with T2DM. The increase in IL-11 levels is associated with decreased pro-apoptotic Bcl-2-associated X protein (BAX) protein production in obese patients with and without T2DM.

## 1. Introduction

More than two billion obese people are registered worldwide [1]. According to the latest data, by 2040, the number of patients with diabetes is projected to increase to 642 million people—10% of the world’s population [1]. Almost 95% of them are patients with (T2DM) [2]. Complications from carbohydrate metabolism are caused by the chronic subclinical inflammation, an imbalance in redox processes, overexpression of reactive oxygen species (ROS), and oxidative stress.

Interleukin (IL)-6 is one of the main cytokines of chronic subclinical inflammation and is a cytokine with multidirectional effects. IL-6 promotes the insulin resistance (IR) and impairs glucose homeostasis [3,4]. Strong experimental data support the ability of IL-6 to potentiate IR in hepatocytes, while the results for adipocytes and skeletal muscle are not always consistent [5,6].

The IL-6 family cytokines and IL-11 belong to the same family, because each cytokine’s receptor complex contains two subunits of the signaling receptor gp130 [6].

The IL-6 family regulates the processes of liver damage/regeneration and maintains the balance between regulatory and effector T cells [6]. Chronic inflammatory processes lead to fatty infiltration of the liver and the spread of foci of inflammation (cellular infiltration) in the parenchyma. In this regard, the role of IL-6 in the liver is of interest in obesity.

A decrease in local and systemic inflammation can be due to the regulation of mitochondrial dynamics and associated processes, such as the activation of cell differentiation and regulation of glucose oxidation processes. Under the influence of oxidative stress, mitochondria adapt by regulating the processes of division/fusion and optimizing mtDNA matrix processes and their biogenesis, thereby exerting a wide influence on metabolic status [7]. Mitochondrial biogenesis is achieved through the organized induction of several transcriptional regulators, the activity of which is critically determined by the prevailing energy requirements [8]. Peroxisome proliferator-activated gamma coactivator (PGC)-1α, (TFAM) [9], and nuclear respiratory factor 1 (NRF-1) are important regulators of mitochondrial biogenesis, and their activity is regulated by post-translational modifications that fine-tune mitochondrial metabolism.

Mitochondrial mechanisms of control of cellular bioenergetic homeostasis require a close connection with the nucleus (interaction between the mitochondrial and nuclear genome) and specifically include mechanisms related to antioxidant defense, protein homeostasis (import, folding, and degradation of proteins through the mitochondrial unfolded protein response), and autophagy for mitochondrial metabolism through the process of mitophagy [7].

Mitophagy is a mitochondria-specific analogue of the autophagy process. The destruction of damaged and dysfunctional mitochondria occurs through autophagy. Mitophagy is an important process for maintaining mitochondrial quality and insulin sensitivity [10]. The fusion or elongation of mitochondria allows the exchange of their contents, mtDNA, and metabolic intermediates and the restoration of the activity of damaged or depolarized membranes [10].

Fusion is predominantly controlled by mitofusins 1 and 2 (Mfn1 and Mfn2) located on the outer mitochondrial membrane, and optic atrophy protein 1 (Opa1) located on the inner mitochondrial membrane [11]. Division or fragmentation increases the number of mitochondria and prepares the cell for division. Division is regulated by fission protein 1 (FIS1), which is transported to the outer mitochondrial membrane dynamin-related protein (Drp) 1 during the process of division initiation [11]. The dynamic transition of mitochondria between fused and fragmented states allows them to reorganize their structure and eliminate damaged or dysfunctional components using mitophagy mechanisms. Cells exposed to a nutrient-rich environment tend to keep their mitochondria in a fragmented state (fission). However, under conditions of starvation and nutrient depletion, mitochondria tend to remain bound or elongated for longer periods of time (fusion).

The fusion and fission processes continuously balance each other: inactivation of one activates the other, and vice versa [7]. In general, these dynamic events regulate mitochondrial function. They carry out mitochondrial distribution, shape, and quality control.

Collectively, liver mitochondria temporarily adapt to increased lipid availability by increasing their oxidative capacity [11]. Loss of mitochondrial adaptation contributes to lipid deposition, ROS accumulation, and, as a consequence, IR. Finally, excessive lipid overload disrupts the cell’s antioxidant capacity and energy balance. This leads to damage to various cell systems, in particular, mitochondrial biogenesis. As a result, IR is aggravated, and steatohepatitis develops.

In this regard, the study aimed to investigate the role of the IL-6 cytokine family (sIL-6Ra, gp130/sIL-6Rb, and IL-11) in the regulation of mitochondrial dynamics in the liver of obese patients and to assess the contribution of these cytokines to the pathogenesis of T2DM.

## 2. Materials and Methods

This study included 134 obese patients. Of these, 70 obese patients had T2DM (48.9 ± 7.28 years; 50.62 ± 8.83 kg/m^2^; 31 men and 39 women), and 115 obese patients did not have carbohydrate metabolism disorders (43.7 ± 9.4 years; 43.8 ± 7.2 kg/m^2^; 52 men and 63 women). The presence of arterial hypertension was noted in 41% of patients. The control group included 41 apparently healthy donors with normal anthropometric and biochemical parameters (39.1 ± 10.1 years; 22.6 ± 3.1 kg/m^2^; 23 men and 18 women). Liver samples were taken during routine laparoscopic operations from conditionally healthy donors: inguinal hernia on the right or left, femoral, diaphragmatic and ventral hernias, and nephroptosis.

All the study participants provided informed consent to participate in a research study. The study was carried out according to the World Medical Association (WMA) Declaration of Helsinki (2000) and the Protocol to the Convention on Human Rights and Biomedicine (1999). The Local Ethics Committee approved the study protocol of the Innovation Park of the Immanuel Kant Baltic Federal University (Protocol No. 4 from 23 October 2013).

The analysis of biochemical parameters in the blood serum was carried out on a Furuno CA-180 biochemical analyzer (Furuno Electric Company, Hyogo, Japan) using DiaSys test systems (DiaSys Diagnostic Systems, Holzheim, Germany). Plasma hormone levels were assessed by flow fluorimetry (Bio-Plex Protein Assay System, Bio-Rad, Heracles, CA, USA) using Bio-Plex Pro™ Human Inflammation Panel 1 test systems, 37-Plex (Bio-Rad, Heracles, CA, USA).

Total RNA from liver biopsies was isolated with ExtractRNA reagent (Evrogen, Moscow, Russia). Reverse transcription of total RNA samples was conducted with the MMLV RT kit (Evrogen, Moscow, Russia). Real-time PCR was conducted on a CFX96 Touch Real-Time PCR Detection System (Bio-Rad, Heracles, CA, USA). PCR data processing was performed using REST 2009 (QIAGEN, Valencia, CA, USA).

Semi-quantitative determination of proteins by immunoblotting was conducted using specific monoclonal antibodies (Thermo Fisher, Waltham, MA, USA) and the Mini-PROTEAN^®^ Tetra Cell Systems and Mini Trans-Blot^®^Turbo blotting systems (Bio-Rad, Heracles, CA, USA). Total protein was isolated from liver biopsies by RIPA buffer (Thermo Fisher, Waltham, MA, USA). The Bradford method was used to measure the total protein concentration (Pierce BCA Protein Assay Kit, Thermo Fisher, Waltham, MA, USA). The specific proteins were detected on a ChemiDoc MP Imaging System (Bio-Rad, Heracles, CA, USA). Densitometry was performed using ImageLab software (Bio-Rad, Heracles, CA, USA).

Paraffin slices of liver biopsies were stained with haematoxylin–eosin. Traditional histological examination was conducted using a Leica DM3000 microscope (Leica Microsystems, Weitzlar, Germany). Additionally, the degree of steatosis and lymphocyte infiltration were assessed using a Pannoramic 250 FLASH (3DHISTECH, Hungary, Budapest) scanning microscope and ImageJ software.

Verification of the normality of quantitative indicator distribution was carried out using the Shapiro-Wilk test. Because the investigated samples fitted a normal distribution, the hypothesis of the equality of the mean sample values was verified using Student’s *t*-tests. To assess the significance of differences between independent quantitative samples that did not follow a normal distribution law, the non-parametric Kruskal-Wallis test was used. To detect statistically significant differences between groups, a pairwise analysis was performed using the non-parametric Mann-Whitney U test for independent groups.

Correlations between the studied indices were determined using Pearson correlation analysis. Differences were considered significant at the level of *p* < 0.05. All statistical analyses were performed in R language (version 3.3.1).

## 3. Results

### 3.1. Biochemical Parameters

The results of the analysis of biochemical parameters of blood are presented in Table 1 and Table 2. In obese patients with T2DM, lipid metabolism disorders were revealed. In this category of patients, the levels of cholesterol, high-density lipoproteins (HDL), low-density lipoproteins (LDL), and triglycerides exceeded the reference values. The cholesterol level of all patients was higher than the control value (*p* < 0.05); therefore, this level in obese patients with T2DM exceeded the value in patients without T2DM (Table 1) (*p* < 0.05). Additionally, in all obese patients, the levels of triglycerides and LDL in the serum significantly exceeded the control values, while the triglyceride level reached maximum values in the group of patients with T2DM (Table 1) (*p* < 0.05).

The test values of alanine aminotransferase (ALT) and aspartate aminotransferase (AST) in obese patients did not differ from those in controls. The level of direct bilirubin was increased in obese patients with and without T2DM, but an increase in total bilirubin was noted only in patients without T2DM (*p* < 0.05) (Table 2).

In the obesity group with T2DM, the glucose content, as expected, was higher than that in the obesity group without T2DM and in the control group (*p* < 0.001) (Table 2).

Thus, in all obese patients, the levels of cholesterol, triglycerides, LDL, and glucose in the serum were significantly higher than the control values.

### 3.2. Inflammatory Mediators

#### Pro-Inflammatory Mediators

In our study, the plasma level of IL-6 in obese patients with T2DM [5.91 (4.45–7.16) pg/mL] was higher than in obese patients without T2DM [3.71 (2.68–4.41) pg/mL] and higher than in controls [1.45 (0.55–6.59) pg/mL] (*p* < 0.01) (Table 3). C-reactive protein (CRP) is an inflammatory biomarker and is an acute phase protein. In our study, the CRP levels in obese patients with T2DM [8.70 (4.2–13.9) mmol/l] and in obese patients without T2DM [10.2 (8.25–16.10) mmol/L] were higher than the control value (*p* < 0.05), which indicates the presence of systemic inflammation in these categories of patients (Table 3).

**Figure 1:**
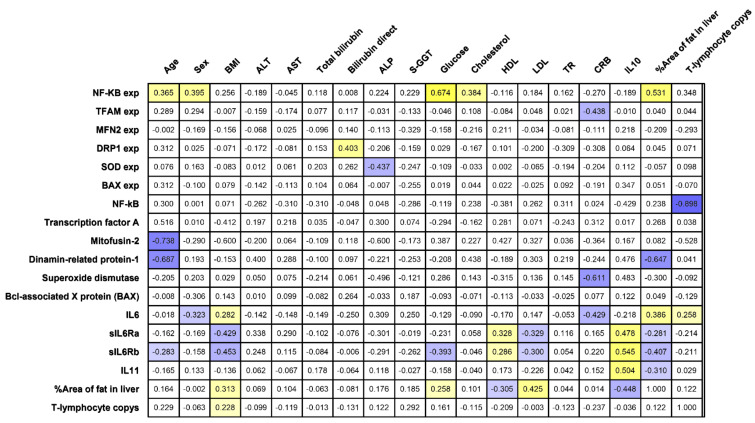
Correlations between the studied parameters in obese patients. Significant positive correlations are indicated in yellow; significant negative correlations are indicated in blue.

Plasma levels of CRP and IL-6 correlated (*r* = −0.429) with the degree of obesity (*r* = 0.282) and suggested that IL-6 is associated with developing metabolic diseases. IL-6 was positively correlated with BMI in obese patients, regardless of the state of carbohydrate metabolism (*r* = 0.282, *p* < 0.05) (Figure 1).

To detail the contribution of IL-6 to the T2DM in obese patients, we studied the plasma level of soluble forms of IL-6 receptors. In our study, the plasma levels of sIL-6Ra [10,222.24 (4919.39–15,021.40) pg/mL] and gp130/sIL-6Rb [38,852.52 (24,600.59–53,741.86) pg/mL] were increased in obese patients without T2DM compared to obese patients with T2DM and controls (*p* < 0.05). sIL-6Ra and gp130/sIL-6Rb plasma levels were four-fold higher in group 2 compared with group 3 (Table 3). This indicates a potential protective role of high plasma levels of sIL-6Ra and gp130/sIL-6Rb receptors with IL-6 in obese patients without T2DM.

The role of IL-6 receptors is characterized in more detail by correlations in obese patients: the levels of soluble receptors sIL-6Ra and gp130/sIL-6Rb were negatively correlated with BMI (*r* = −0.43, *p* < 0.05) (*r* = −0.45, *p* < 0.05) and with the LDL level (*r* = −0.33, *p* < 0.05) (*r* = −0.30, *p* < 0.05) and positively correlated with the HDL level (*r* = 0.33, *p* < 0.05) (*r* = 0.29, *p* < 0.05) (Figure 1). This highlights the potential protective role of sIL-6Ra and gp130/sIL-6Rb in maintaining normal lipid balance in obese patients. The sIL-6Ra and gp130/sIL-6Rb receptors were positively correlated with each other and with IL-10. An effect on carbohydrate metabolism was noted: the level of sIL-6Ra, but not the level of gp130/sIL-6Rb, negatively correlated with the level of glucose (*r* = −0.29, *p* < 0.05). This shows that the sIL-6Ra receptor has a more substantial effect on carbohydrate metabolism.

**Figure 2:**
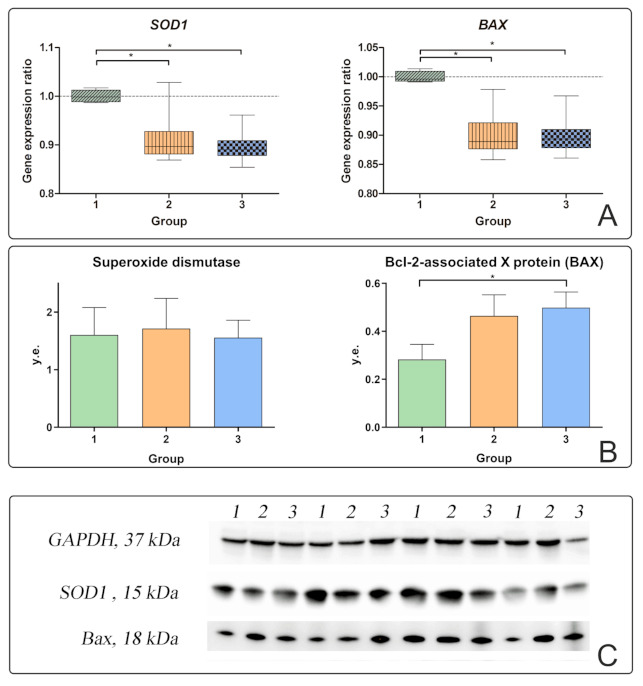
Characteristics of apoptotic processes in the liver in obese patients with and without T2DM. 1—control group; 2—obesity group without T2DM; 3—obesity group with T2DM; (**A**) gene expression ratio of *SOD1* and *BAX* in liver biopsies; (**B**) production of superoxide dismutase (SOD1) and Bcl-2-associated X protein (BAX) proteins in liver biopsies; (**C**) Western blot analysis of liver biopsies. The significance was determined using the Mann–Whitney criterion for two independent samples (* *p* < 0.05).

IL-11 was increased in obese patients without T2DM compared to obese patients with T2DM and controls. The IL-11 level was positively correlated with IL-10, which may indicate its compensatory enhancement in T2DM. The IL-11 level was negatively correlated with the production of BAX protein in the liver (Figure 2B).

### 3.3. Investigation of the Inflammation Processes in Liver Biopsies

**Figure 3:**
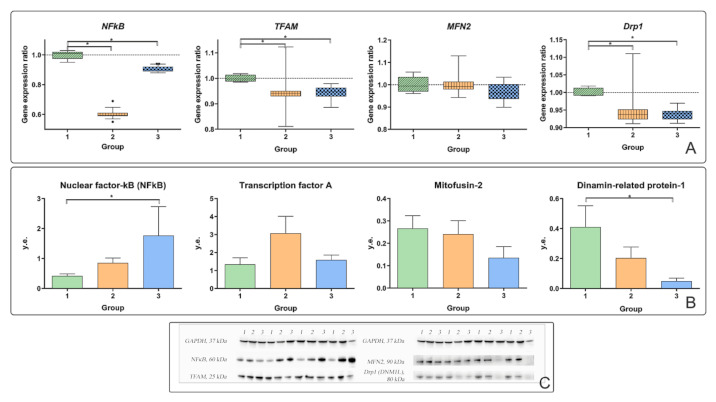
Characteristics of the processes of mitochondrial dynamics in the liver of obese patients with and without T2DM. 1—control group; 2—obesity group without T2DM; 3—obesity group with T2DM; (**A**) gene expression ratios of *NFkB, TFAM, MFN2* and *DRP1* in liver biopsies; (**B**) nuclear factor-kB (NFkB), transcription factor A (TFAM), mitofusin (MFN2) and dinamin-related protein-1 (DRP1) protein production in liver biopsies; (**C**) Western blot analysis of liver biopsies. The significance was determined using the Mann–Whitney criterion for two independent samples (* *p* < 0.05).

We investigated the expression of genes and proteins in liver biopsies to characterize liver tissue inflammation in obese patients. The nuclear factor kappa B (*NFkB*) gene expression ratio in liver biopsies was higher in obese patients with T2DM than in obese patients without T2DM (Figure 3A). However, the *NFkB* gene expression ratio in liver biopsies was significantly lower than that in the controls. In contrast, *NFkB* protein production in liver biopsies was higher in patients with T2DM (Figure 3B). The *NFkB* gene expression ratio in liver biopsies was positively correlated with the concentrations of cholesterol, glucose, and age in all obese patients.

IL-6 and its receptors are associated with inflammatory processes in the liver in obesity. Plasma levels of sIL-6Ra and gp130/sIL-6Rb are negatively correlated with the *NFkB* gene expression ratio in liver biopsies of obese patients. Negative correlations may indicate a compensatory enhancement of IL-6 receptors in the development of inflammation in the liver.

Next, we tested whether the receptors are associated with antioxidant systems in the liver. A negative correlation of IL-6 in plasma was found with *SOD1* gene expression ratio in all obese patients (r = −0.81) (Figure 2A). The *SOD1* gene expression ratios in the liver decreased in obese patients with and without T2DM relative to the controls. SOD1 protein production was not associated with any of the investigated factors. Thus, the compensatory increases in IL-6 receptors were realized without superoxide dismutase.

In patients with T2DM, the BAX pro-apoptotic gene expression ratio decreased and BAX protein production increased relative to the controls (Figure 2A,B). This may indicate the cessation of protein synthesis and its activity directly in liver tissues. BAX protein production in liver biopsies was positively correlated with *NFkB* protein production in liver biopsies (Figure 1). Inflammatory processes in liver tissue stimulated apoptosis of liver cells.

The processes of induction of apoptosis in the liver are inhibited by another cytokine from the IL-6 family, IL-11. The highest level of IL-11 was recorded in patients without T2DM and was negatively correlated with BAX protein production in liver biopsies of obese patients.

### 3.4. Studies of Liver Biopsies

**Figure 4:**
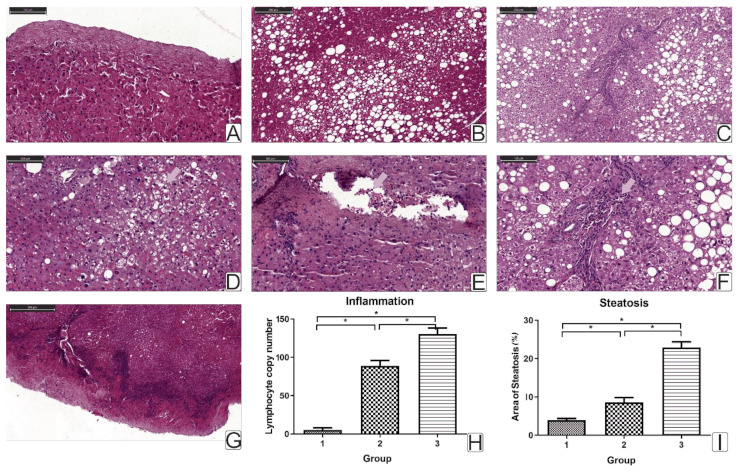
Histological study of liver biopsies. (**A**) Liver capsule of a healthy donor; (**B**) small, medium, and large droplet steatosis. Magnification 20×; (**C**) lymphocytic infiltration in the portal tract, which further spreads into the tissue to the foci of steatosis. Intracellular cholestasis and signs of dystrophy and karyolysis. In the foci of steatosis, there are large, medium, and small droplet inclusions. Magnification 10×; (**D**) intracellular cholestasis. Magnification 20×; (**E**) lymphocytic infiltration in the portal tracts. Magnification 20×; (**F**) lymphocytic infiltration in the portal tract, which further spreads into the tissue to the foci of steatosis. Intracellular cholestasis and signs of dystrophy and karyolysis. In the foci of steatosis, there are large, medium, and small droplet inclusions. Magnification 20×; (**G**) liver capsule with inflammation; (**H**) the number of lymphocytes in liver biopsies. 1—control group, 2—obesity group without T2DM, 3—obesity group with T2DM; (**I**) comparison of the area of fat inclusions of steatosis. 1—control group, 2—obesity group without T2DM, 3—obesity group with T2DM. The significance was determined using the Mann–Whitney criterion for two independent samples (* *p* < 0.05).

The control group showed normal liver morphology without signs of inflammation. However, some patients had focal steatosis (small and medium droplets), as well as sporadic fatty inclusions (Figure 4A).

In the obesity group without T2DM, small-, medium- and large-droplet steatosis was observed (Figure 4B). Manifestations of hepatocyte dystrophy and karyolysis were found in 40% of patients (17%) (Figure 4C). In 41.6% of patients, intracellular cholestasis was detected (Figure 4C,D). Signs of inflammation (lymphocyte infiltration) were found in 58% of patients (Figure 4C,E). Meanwhile, 30% had no steatosis or had single droplets as in the control group.

In the obesity group with T2DM, all patients had small-, medium- and large-droplet steatosis. Dystrophy of hepatocytes was observed in almost all patients (93.75%), with 62.5% manifesting with karyolysis (Figure 4F, magn.). Intracellular cholestasis, lymphocytic infiltration, and enlarged liver capsules were detected in 81.25% of patients (Figure 4G).

In patients with T2DM, signs of NAFLD were recorded, and steatosis and steatohepatitis were diagnosed. Steatohepatitis is distinguished from steatosis by the presence of an immune cell leukocyte infiltrate. According to the results of the histological study of liver biopsies, an increase in the area of fatty deposits and the number of lymphocytes was found in obese patients with T2DM. These facts characterize the presence of the pro-inflammatory component at the local (liver biopsy) and systemic (peripheral blood) levels in T2DM. In addition, as mentioned earlier, in patients with T2DM, an increase in pro-inflammatory factors (in the circulation—IL-6 and CRP (Table 3); in liver biopsies—the production of NFkB was revealed compared to patients without T2DM (Figure 3A).

Observed in a prevailing number of patients with T2DM, NAFLD with a predominance of steatohepatitis is a factor in the change in mitochondrial biogenesis in the liver. On the other hand, changes in mitochondrial biogenesis parameters potentially contribute to the progression of steatohepatitis. To search for the relationship between the inflammatory components and mitochondrial dynamics, we investigated the expression of genes and the production of proteins responsible for these processes in liver biopsies.

### 3.5. Mitochondrial Dynamics

In patients with T2DM, the *TFAM* gene expression ratio decreased relative to the controls. In patients with T2DM, the *DRP1* gene expression ratio and DRP1 protein production were reduced relative to the controls. However, there was a tendency towards an increase in the expression of the *TFAM* and *DRP1* genes and the production of the TFAM protein in some obese patients without T2DM, which is evident from the range of the median in Figure 3A,B. In patients with T2DM, replication, transcription and division in liver biopsies were reduced. This could be a sign that mitochondrial dynamics are declining and tend to fuse in inflammatory conditions.

We did not find any significant changes in mitochondrial dynamics in obese patients without T2DM; therefore, we tested the hypothesis about the effect of the microenvironment of chronic subclinical inflammation on the liver in patients with T2DM.

The plasma level of IL-6 was negatively correlated with the production of TFAM protein, which is a mitochondrial transcription factor responsible for the regulation of division and replication. TFAM protein production was independent of other parameters and was associated only with IL-6 levels. IL-6 is able to regulate the processes of replication and transcription of mitochondrial DNA. Considering the critical role of IL-6 and its receptors in the regulation of lipid metabolism, its effect on fatty liver infiltration deserves special attention.

We examined the effect of anthropometric parameters on mitochondrial dynamics. Age is an important risk factor, and mitochondrial dynamics decreased with age. The levels of DRP-1 and MFN2 proteins were negatively correlated with age.

Thus, mitochondrial dynamics in liver biopsies of obese patients were influenced by age and the levels of IL-6 in the plasma.

## 4. Discussion

The liver is exposed to higher levels of nutrients from food than peripheral tissues [12]. Nutrients penetrate the liver the fastest through the hepatic portal vein [13]. Hepatocytes can store and release glucose to minimize pre/post-meal imbalances [14].

In addition to carbohydrate metabolism, the liver plays an important role in lipid, protein, and pigment metabolism [13]. Lipid metabolism disorders were typical for patients with T2DM and contributed to liver diseases in our study. The liver’s excretory function is bile secretion, which includes direct bilirubin, creatinine, urea, and cholesterol [13]. The total bilirubin level was increased in obese patients with and without T2DM, indicating abnormalities in the hepatic parenchyma.

Obesity, inflammation in adipose tissue, and IR are closely related to each other, forming a vicious circle that leads to steatosis [15,16]. Various serum factors in the blood of subjects with obesity induce inflammation and oxidative stress through various mechanisms and can negatively affect insulin signaling [16]. We measured the levels of pro-inflammatory factors involved in the development of chronic subclinical inflammation. CRP and IL-6 levels in obese patients with T2DM were elevated in plasma and indicated systemic inflammation in this patient population. In obese patients with T2DM, CRP levels were lower than those in obese patients without T2DM. This is unusual; other authors have shown no difference in CRP levels between patients with steatosis and steatohepatitis [17]. CRP is synthesized in the liver, and its decrease in patients with T2DM may result from liver dysfunction 130.

Most obese patients have been diagnosed with steatosis. In obese patients with T2DM, steatosis was higher than that in obese patients without T2DM. Steatosis leads to increased NFkB signaling through the activation of an inhibitor of nuclear factor-κB kinase β (IKKβ) [18]. NFkB activation induces pro-inflammatory mediator production. Studies in mice have shown that activation of hepatocyte inflammation may be a link between initial metabolic stress and the death of hepatocytes and stimulation of fibrogenesis in steatohepatitis [19]. In patients with T2DM, the production of NFkB protein in the liver was higher than that in control patients (Figure 3B).

Hepatic inflammation in obesity leads to the progression of steatosis and steatohepatitis. We have shown that chronic inflammatory processes in obese patients lead to steatosis and liver lymphocyte infiltration. A study of liver biopsy histology showed an increase in the number of lymphocytes in obese patients with T2DM.

The percent steatosis and liver lymphocyte infiltration were associated with BMI (*r* = 0.31, *r* = 0.29, *p* < 0.05) and with the level of plasma IL-6 (*r* = 0.39, *r* = 0.26, *p* < 0.05) (Figure 1). The area of fat inclusions was positively correlated with the levels of glucose (r = 0.25) and LDL (*r* = 0.425) and negatively correlated with HDL (*r* = −0.30) (*p* < 0.05) in all obese patients, which formed a vicious circle with a positive inverse link. These facts characterize a pro-inflammatory component at the local (liver biopsy) and systemic (peripheral blood) levels in T2DM.

IL-6 binds to IL-6R or sIL-6R and initiates subsequent signaling through interaction with gp130 [5,6]. The IL-6/sIL-6R complex acts as an agonist of gp130-mediated IL-6 signaling and is called trans-signaling. This type of signaling broadens the spectrum of potential targets for IL-6 to virtually any cell type due to the ubiquitous expression of gp130. However, gp130 can also be found in a soluble form, which results in the inhibition of trans-IL-6 signaling while not affecting the classic type of signaling [6]. Soluble gp130 (sgp130) is found in healthy people’s bloodstream, inhibiting the circulating IL-6 complex’s systemic response with sIL-6Ra [6,20,21,22].

Plasma sIL-6Ra and gp130/sIL-6Rb increased in obese patients without T2DM compared with obese patients with T2DM and controls. Both sIL-6Ra and gp130/sIL-6Rb receptors negatively correlated with BMI (*r* = −0.43, *p* < 0.05) (*r* = −0.45, *p* < 0.05) and LDL levels (*r* = −0.33, *p* < 0.05) (*r* = −0.30, *p* < 0.05) and positively correlated with HDL levels (*r* = 0.33, *p* < 0.05) (*r* = 0.29, *p* < 0.05). This emphasizes the protective role of compensatory increased sIL-6Ra and gp130/sIL-6Rb in maintaining normal lipid balance in obese patients. In patients without T2DM, fat infiltration hepatocytes were found in a much smaller number of cases than in patients with T2DM.

Classic IL-6 signaling in T cells plays a key role in the early stages of obesity, while trans-signals are more important later. Mice with hepatocyte-specific IL-6Rα deletion exhibit decreased insulin sensitivity and glucose tolerance, indicating a potential protective role for hepatic IL-6 signaling [23]. Sgp130Fc can block trans-signaling IL-6 without affecting classical signaling. Therefore, future IL-6-based therapies may use a specific blockade of trans-IL-6 signaling rather than a global blockade of all IL-6 activities [22]. The blockade of trans-IL-6 signals was sufficient to suppress the inflammatory state [24]. Moreover, these data demonstrate the importance of sIL-6R levels for the pathophysiology of inflammatory diseases.

The anti-inflammatory and regenerative properties of IL-6 are mediated by a classical signaling mode, while pro-inflammatory responses in many pathological conditions involve trans-signaling.

Classic IL-6 signaling occurs via IL-6R and stimulates liver regeneration, protecting the liver from damage. An increase in plasma IL-6 levels leads to lipolysis in skeletal muscle and systemic fatty acid oxidation [6]. Infusion of IL-6 into human AT enhances fatty acid and glycerol uptake, which promotes lipolysis. IL-6 suppressed CRP production. Plasma IL-6 levels were negatively correlated with CRP (*r* = −0.43, *p* < 0.05).

Activation of classic IL-6 signaling in steatosis leads to increased proliferation of liver cells and the replication stress due to the accumulation of replication errors, contributing to the progression of pathogenesis and degeneration of hepatocytes. In the group of obese patients with T2DM, almost all patients had dystrophy of hepatocytes and karyolysis manifestations; manifestations of intracellular cholestasis and lymphocytic infiltration of patients were noted. Oxidative stress in patients with NAFLD leads to the death of hepatocytes [18,20]. In patients with T2DM, the production of pro-apoptotic BAX protein increased together with the production of NFkB. This proves that hepatic inflammation stimulates liver cell apoptosis. Liver cells infiltrated with fatty inclusions die through various mechanisms, including apoptosis, necrosis, and necroptosis. In an attempt to regenerate new cells, NASH progresses to fibrosis and cirrhosis with hepatocytes replaced by scar tissue collagen type 1 produced by stellate cells [18].

The IL-6 begins to be synthesized in response to liver damage. The feedback mechanism requires more detailed study. In our study, no correlations of IL-6 in blood plasma with any characteristics of apoptosis were found. However, a negative correlation of IL-6 in blood plasma was found with the level of *SOD1* gene expression in the liver in all obese patients (*r* = −0.815). The level of *SOD1* gene expression in the liver decreased relative to the controls in obese patients with and without T2DM, which indicates a low antioxidant activity of the liver tissue. SOD protein production was not associated with any of the investigated factors. Against the background of an increase in plasma IL-6 levels and the development of liver cell inflammation and death, superoxide dismutase ceases to inhibit steatohepatitis in patients with T2DM.

IL-11 inhibits the induction of apoptosis in the liver. The highest levels of IL-11 were found in patients without T2DM and were negatively correlated with the production of BAX protein in liver biopsies of obese patients. IL-11 has been implicated in fibrosis of the heart [25], liver [12], and lung [26]. Blocking IL-11 is considered a promising target for the fight against liver disease, with an established mechanism of action in both acute and chronic liver diseases [27].

IL-11 is increased in obese patients without T2DM compared with patients with T2DM and controls. The IL-11 level is also positively correlated with IL-10, which may indicate its protective role in T2DM. However, IL-11 was not associated with lipid metabolism. Plasma IL-11 levels were negatively correlated with BAX protein production in liver biopsies. IL-11 is also soluble [22,28] and is blocked by sgp130 binding. How sgp130 affects the pathways triggered by these cytokines and how these pathways influence the effects of sgp130 deserve further study.

Strengthening mitochondrial dynamics is associated with increased cell proliferation during apoptosis. We did not find any significant changes in mitochondrial dynamics in obese patients without T2DM; therefore, we tested the hypothesis about the effect of the microenvironment of chronic subclinical inflammation on the liver in patients with T2DM. In obese patients, mitochondrial dysfunction, mediated by an increase in TFAM in the presence of an inflammatory process in the liver, may be a factor contributing to the formation of T2DM.

In this case, oxidative stress can cause changes in mtDNA. mtDNA is especially sensitive to oxidative damage because it is directly adjacent to ROS production and DNA repair systems. In addition, oxidative damage to nuclear DNA can lead to mitochondrial dysfunction by disrupting the transcription of nuclear-encoded mitochondrial genes. For example, the expression levels of genes involved in mitochondrial metabolism and biogenesis, such as *TFAM*, *NRF-2*, and *PGC-1α*, are reduced in NAFLD [29].

Plasma IL-6 suppresses the production of TFAM protein in liver biopsies (*r* = −0.615, *p* < 0.05). TFAM is a mitochondrial transcription factor responsible for the regulation of division and replication. TFAM protein production was independent of other parameters and was associated only with IL-6 levels. IL-6 can regulate the processes of replication and transcription of mitochondrial DNA. Considering the important role of IL-6 and its receptors in regulating lipid metabolism, its effect on fatty liver infiltration deserves special attention. TFAM is a multifunctional DNA-binding protein required for the activation of transcription and organization of mtDNA [29]. TFAM is one of the key regulators of mtDNA transcription associated with altered mitochondrial division/fusion processes and mtDNA replication [29]. Thus, in patients with T2DM, an increase in TFAM transcription and a decrease in its protein level were observed in in vitro models [29]. The TFAM protein is synthesized in the cell and transported into mitochondria, where it activates transcription and participates in mtDNA replication [29]. In obese patients with T2DM, the level of *TFAM* gene expression and TFAM protein production increased in some patients, but these changes were not significant. The *TFAM* gene plays an important role in cell physiology, participates in mtDNA maintenance, and regulates the number of mtDNA copies [29]. Therefore, an increase in the expression level of the *TFAM* gene may indicate an increase in mtDNA transcription under conditions of inflammation of the liver tissue. The expression level of the *TFAM* gene was positively correlated with the expression level of the *DRP1* (*r* = 0.62) and *SOD1* (*r* = 0.53) genes.

Proteins that regulate the fusion and division of mitochondria are mainly responsible for changing the dynamics of mitochondria: MFN1, MFN2, DRP1, and FIS1. MFN2 is required for mitochondrial fusion, while DRP1 is required for mitochondrial division [29,30]. Mitofusin is located on the outer mitochondrial membrane and promotes fusion of the outer mitochondrial membranes. In addition to its role in mitochondrial fusion, mitofusin may play a role in developing oxidative stress during metabolic syndrome. For the division processes, one of the main proteins is DRP1. DRP1 is found mainly in the cytosol and is recruited to the outer mitochondrial membrane by the FIS1 protein located on the outer membrane.

In obese patients with T2DM, the level of gene expression and production of MFN2 and DRP1 proteins decreased. In obese patients with T2DM, the expression of the *DRP1* gene and the production of the DRP-1 protein were reduced relative to the controls (*p* < 0.05) (Figure 3A,B).

It is assumed that mitochondrial fusion processes are induced under conditions that require the optimization of mitochondrial bioenergetics. At the same time, the processes of division are associated with the degradation of mitochondria. Consequently, the processes of fusion and division are induced under conditions of mitochondrial damage. Repression of MFN2 is associated with a decrease in cellular metabolism. However, no changes in MFN2 were found in our study.

Drp1 is also involved in the regulation of apoptosis [31]. Mitophagy, a specialized form of macroautophagy, mediates selective degradation of damaged mitochondria [7]. In response to reduced membrane potential, ubiquitin is activated in mitochondria, removing Mfn and several other outer mitochondrial membrane proteins. Ultimately, this leads to the capture of mitochondria by the autophagosome and subsequent catabolism in the lysosomes [7]. The expression level of the *DRP1* gene was positively correlated with the level of BAX gene expression in the liver (*r* = 0.40, *p* < 0.05). With an increase in inflammation in the liver, the processes of mitophagy and mitochondrial division are activated.

Dysregulation of energy metabolism in insulin-dependent tissues can be a crucial cause of T2DM. With the development of T2DM, the processes of mitochondrial and peroxisomal oxidation are triggered; the latter’s activation is associated with excessive accumulation of ROS and the development of oxidative stress [32]. Under the influence of oxidative stress and increased ROS production, obesity disrupts the functioning of mitochondria, represented by a decrease in the synthesis of ATP [32] by mitochondria, increased mitophagy, and processes of its division. Thus, in liver biopsies of patients with T2DM, replication processes, transcription, and division were reduced.

## 5. Conclusions

Inflammation on liver biopsy is associated with cholesterol and glucose concentrations and age, leading to steatohepatitis progression in obese patients.

Increasing the concentration of sIL-6Ra and gp130/sIL-6Rb protects against carbohydrate disorders in obese patients and prevents NAFLD progression in obese patients.

An increase in plasma IL-6 levels is associated with decreased TFAM protein production in liver biopsies in obese patients with and without type 2 diabetes. Replication, transcription, and division processes in liver biopsy were reduced in patients with type 2 diabetes.

Inflammatory processes stimulate liver cell apoptosis in obese patients with type 2 diabetes. The increase in IL-11 levels is associated with decreased BAX protein production in obese patients with and without type 2 diabetes.

## Figures and Tables

**Table 1:** Basic biochemical parameters of lipid metabolism in obese patients with and without type 2 diabetes mellitus (T2DM).

Groups	Control Group	Obese Patients without T2DM	Obese Patients with T2DM
	1	2	3
Cholesterol (<5.2) mmol/L	4.23 ± 0.99	4.83 ± 1.26 *p*_1__-2_ < 0.05 *	5.20 ± 1.02 *p*_1-3_ < 0.05 * *p*_2-3_ < 0.05 *
Triglycerides (<2.53) mmol/L	1.44 ± 0.81	1.59 ± 0.81 *p*_1-2_ < 0.05 *	2.00 ± 0.87 *p*_1-3_ < 0.05 * *p*_2-3_ < 0.05 *
HDL (0.78–1.81) mmol/L	1.38 ± 0.86	1.18 ± 0.32	1.16 ± 0.53
LDL (0.00–3.4) mmol/L	2.18 ± 0.80	2.87 ± 0.88 *p*_1-2_ < 0.05 *	2.98 ± 0.75 *p*_1-3_ < 0.05 *

Note: * *p* < 0.05, significance was determined using the *t*-test, (mean ± SD). Reference values are indicated in parentheses under the name of the metabolite. *p*_1-2_—differences between groups 1 and 2; *p*_1-3_—differences between groups 1 and 3; *p*_2-3_—differences between groups 2 and 3.

**Table 2:** Basic biochemical parameters of hepatic enzymes in the blood of obese patients with and without T2DM.

Groups	Control Group	Obese Patients without T2DM	Obese Patients with T2DM
	1	2	3
ALT (<41) U/L	23.46 ± 15.98	19.54 ± 10.14	31.42 ± 16.50
AST (<35) U/L	17.57 ± 4.15	20.87 ± 5.31	23.20 ± 9.09
Alkaline phosphatase (<258) U/L	167.21 ± 77.21	178.68 ± 48.52	194.86 ± 52.84 *p*_1-3_ < 0.05 *
Gamma-glutamyl transpeptidase (<49) U/L	21.75 ± 9.24	24.81 ± 14.26	39.27 ± 15.61 *p*_1-3_ < 0.05 * *p*_2-3_ < 0.05 *
Total bilirubin (1.7–21) mmol/L	10.26 ± 3.84	13.13 ± 9.45 *p*_1-2_ < 0.05 *	11.5 ± 4.81
Direct bilirubin (0.00–3.4) mmol/L	1.61 ± 1.07	2.86 ± 2.01 *p*_1-2_ < 0.05 *	2.88 ± 1.52 *p*_1-3_ < 0.05 *
Glucose (3.9-6.4) mmol/L	4.98 ± 1.19	5.28 ± 1.60	8.43 ± 2.75 *p*_1-3_ < 0.001 ** *p*_2-3_ < 0.001 **

Note: * *p* < 0.05, ** *p* < 0.001, significance was determined using the *t*-test (mean ± SD). Reference values are indicated in parentheses under the name of the metabolite. *p*_1-2_—differences between groups 1 and 2; *p*_1-3_—differences between groups 1 and 3; *p*_2-3_—differences between groups 2 and 3.

**Table 3:** Basic biochemical parameters of liver enzymes in obese patients with and without T2DM.

Groups	Control Group	Obese Patients without T2DM	Obese Patients with T2DM
	1	2	3
IL-6 (pg/mL)	1.45 (0.55–3.39)	3.71 (2.68–4.41) *p*_1-2_ = 0.04 *	5.91 (4.45–7.16) *p*_1-3_ < 0.01 * *p*_2-3_ < 0.01 *
sIL-6Ra (pg/mL)	2899.16 (2192.10–5856.15)	10222.24 (4919.39–15021.40) *p*_1-2_ < 0.01 *	2235.62 (1077.12–3156.69) *p*_1-3_ = 0.01 * *p*_2-3_ < 0.01 *
gp130/sIL-6Rb (pg/mL)	13,899.75 (10917.07–29468.43)	38852.52 (24600.59–53741.86) *p*_1-2_ < 0.01 *	4479.41 (2338.51–5646.27) *p*_1-3_ < 0.01 * *p*_2-3_ < 0.01 *
IL-10 (pg/mL)	0.64 (0.32–1.53)	2.18 (1.11–3.44) *p*_1-2_ < 0.01 *	0.75 (0.26–0.99) *p*_2-3_ < 0.01 *
IL-11 (pg/mL)	0.18 (0.12–0.48)	0.94 (0.52–1.47) *p*_1-2_ < 0.01 *	0.50 (0.40–0.69) *p*_1-3_ < 0.01 * p_2-3_ < 0.01 *
CRP (mmol/l)	3.96 (1.56–8.83)	10.20 (8.25–16.10) *p*_1-2_ < 0.01 *	8.70 (4.20–13.90) *p*_1-3_ = 0.01 *

Note: The significance was determined using the Mann–Whitney criterion for two independent samples (* *p* < 0.05). *p*_1-2_—differences between groups 1 and 2; *p*_1-3_—differences between groups 1 and 3; *p*_2-3_—differences between groups 2 and 3.

## Data Availability

The data are available upon request from the author’s correspondents.

## References

[1] Obesity and Overweight. https://www.who.int/news-room/fact-sheets/detail/obesity-and-overweight.

[2] Statistics about Diabetes|ADA. https://www.diabetes.org/resources/statistics/statistics-about-diabetes.

[3] Sarbijani H.M., Khoshnia M., Marjani A. (2016). The association between Metabolic Syndrome and serum levels of lipid peroxidation and interleukin-6 in Gorgan. Diabetes Metab. Syndr..

[4] Bastard J.P., Jardel C., Bruckert E., Blondy P., Capeau J., Laville M., Vidal H., Hainque B. (2000). Elevated levels of interleukin 6 are reduced in serum and subcutaneous adipose tissue of obese women after weight loss. J. Clin. Endocrinol. Metab..

[5] Qu D., Liu J., Lau C.W., Huang Y. (2014). IL-6 in diabetes and cardiovascular complications. Br. J. Pharmacol..

[6] Rose-John S. (2018). Interleukin-6 Family Cytokines. Cold Spring Harb. Perspect. Biol..

[7] Lee J., Park J.-S., Roh Y.S. (2019). Molecular insights into the role of mitochondria in non-alcoholic fatty liver disease. Arch. Pharm. Res..

[8] Thoudam T., Jeon J.-H., Ha C.-M., Lee I.-K. (2016). Role of Mitochondria-Associated Endoplasmic Reticulum Membrane in Inflammation-Mediated Metabolic Diseases. Mediat. Inflamm..

[9] Xie D., Wu X., Lan L., Shangguan F., Lin X., Chen F., Xu S., Zhang Y., Chen Z., Huang K. (2016). Downregulation of TFAM inhibits the tumorigenesis of non-small cell lung cancer by activating ROS-mediated JNK/p38MAPK signaling and reducing cellular bioenergetics. Oncotarget.

[10] Nassir F., Ibdah J.A. (2014). Role of Mitochondria in Nonalcoholic Fatty Liver Disease. Int. J. Mol. Sci..

[11] Pich S., Bach D., Briones P., Liesa M., Camps M., Testar X., Palacín M., Zorzano A. (2005). The Charcot–Marie–Tooth type 2A gene product, Mfn2, up-regulates fuel oxidation through expression of OXPHOS system. Hum. Mol. Genet..

[12] Widjaja A.A., Singh B.K., Adami E., Viswanathan S., Dong J., D’Agostino G.A., Ng B., Lim W.W., Tan J., Paleja B.S. (2019). Inhibiting Interleukin 11 Signaling Reduces Hepatocyte Death and Liver Fibrosis, Inflammation, and Steatosis in Mouse Models of Nonalcoholic Steatohepatitis. Gastroenterology.

[13] Buzzetti E., Pinzani M., Tsochatzis E.A. (2016). The multiple-hit pathogenesis of non-alcoholic fatty liver disease (NAFLD). Metabolism.

[14] Kim O.-K., Jun W., Lee J. (2015). Mechanism of ER Stress and Inflammation for Hepatic Insulin Resistance in Obesity. Ann. Nutr. Metab..

[15] Shulman G.I. (2000). Cellular mechanisms of insulin resistance. J. Clin. Investig..

[16] Tarantino G., Finelli C. (2013). What about non-alcoholic fatty liver disease as a new criterion to define metabolic syndrome?. World J. Gastroenterol. WJG.

[17] Oruc N., Ozutemiz O., Yuce G., Akarca U.S., Ersoz G., Gunsar F., Batur Y. (2009). Serum procalcitonin and CRP levels in non-alcoholic fatty liver disease: A case control study. BMC Gastroenterol..

[18] Tiniakos D.G., Vos M.B., Brunt E.M. (2010). Nonalcoholic fatty liver disease: Pathology and pathogenesis. Annu. Rev. Pathol..

[19] Csak T., Ganz M., Pespisa J., Kodys K., Dolganiuc A., Szabo G. (2011). Fatty Acid and Endotoxin Activate Inflammasomes in Mouse Hepatocytes that Release Danger Signals to Stimulate Immune Cells. Hepatology.

[20] Sanyal A.J., Brunt E.M., Kleiner D.E., Kowdley K.V., Chalasani N., Lavine J.E., Ratziu V., McCullough A. (2011). Endpoints and clinical trial design for nonalcoholic steatohepatitis. Hepatology.

[21] Covarrubias A., Horng T. (2014). IL-6 strikes a balance in metabolic inflammation. Cell Metab..

[22] Reeh H., Rudolph N., Billing U., Christen H., Streif S., Bullinger E., Schliemann-Bullinger M., Findeisen R., Schaper F., Huber H.J. (2019). Response to IL-6 trans- and IL-6 classic signalling is determined by the ratio of the IL-6 receptor α to gp130 expression: Fusing experimental insights and dynamic modelling. Cell Commun. Signal..

[23] Zhuang Z., Pan X., Zhao K., Gao W., Liu J., Deng T., Qin W. (2019). The Effect of Interleukin-6 (IL-6), Interleukin-11 (IL-11), Signal Transducer and Activator of Transcription 3 (STAT3), and AKT Signaling on Adipocyte Proliferation in a Rat Model of Polycystic Ovary Syndrome. Med. Sci. Monit. Int. Med. J. Exp. Clin. Res..

[24] Didion S.P. (2017). Cellular and Oxidative Mechanisms Associated with Interleukin-6 Signaling in the Vasculature. Int. J. Mol. Sci..

[25] Schafer S., Viswanathan S., Widjaja A.A., Lim W.-W., Moreno-Moral A., DeLaughter D.M., Ng B., Patone G., Chow K., Khin E. (2017). IL-11 is a crucial determinant of cardiovascular fibrosis. Nature.

[26] Ng B., Dong J., D’Agostino G., Viswanathan S., Widjaja A.A., Lim W.-W., Ko N.S.J., Tan J., Chothani S.P., Huang B. (2019). Interleukin-11 is a therapeutic target in idiopathic pulmonary fibrosis. Sci. Transl. Med..

[27] Widjaja A.A., Chothani S.P., Cook S.A. (2020). Different roles of interleukin 6 and interleukin 11 in the liver: Implications for therapy. Hum. Vaccines Immunother..

[28] Metcalfe R.D., Putoczki T.L., Griffin M.D.W. (2020). Structural Understanding of Interleukin 6 Family Cytokine Signaling and Targeted Therapies: Focus on Interleukin 11. Front. Immunol..

[29] Pohjoismäki J.L.O., Wanrooij S., Hyvärinen A.K., Goffart S., Holt I.J., Spelbrink J.N., Jacobs H.T. (2006). Alterations to the expression level of mitochondrial transcription factor A, TFAM, modify the mode of mitochondrial DNA replication in cultured human cells. Nucleic Acids Res..

[30] Makino A., Scott B.T., Dillmann W.H. (2010). Mitochondrial fragmentation and superoxide anion production in coronary endothelial cells from a mouse model of type 1 diabetes. Diabetologia.

[31] El-Hattab A.W., Craigen W.J., Scaglia F. (2017). Mitochondrial DNA maintenance defects. Biochim. Biophys. Acta Mol. Basis Dis..

[32] Sivitz W.I., Yorek M.A. (2010). Mitochondrial dysfunction in diabetes: From molecular mechanisms to functional significance and therapeutic opportunities. Antioxid. Redox Signal..

